# Direct manipulation of perceived angular declination affects perceived size and distance: a replication and extension of Wallach and O’Leary (1982)

**DOI:** 10.3758/s13414-015-0864-y

**Published:** 2015-03-20

**Authors:** Morgan J. C. Williams, Frank H. Durgin

**Affiliations:** 1 Department of Psychology, Swarthmore College, Swarthmore, Pennsylvania; 2 Department of Psychology, Swarthmore College, 500 College Avenue, Swarthmore, Pennsylvania 19081

**Keywords:** 3-D perception, Space perception, Visual perception, Spatial cognition

## Abstract

In two experiments involving a total of 83 participants, the effect of vertical angular optical compression on the perceived distance and size of a target on the ground was investigated. Replicating an earlier report (Wallach & O’Leary, 1982), reducing the apparent angular declination below the horizon produced apparent object width increases (by 33 %), consistent with the perception of a greater ground distance to the object. A throwing task confirmed that perceived distance was indeed altered by about 33 %. The results are discussed in relation to cue recruitment and to recent evidence of systematic bias in the perception of angular declination.

The rules of pictorial perspective include the implication that the higher the base of a terrestrial object appears in a picture, the farther away it is along the ground (Gibson, 1950), and thus that “height in the field” is a pictorial cue to distance that represents proximity to the horizon (see Sedgwick, 1980). When an observer is situated within a 3-D environment, the precise angular direction to the point at which an object contacts a horizontal ground plane can provide a direct measure of the distance to the object along the ground, provided that one can (implicitly) take one’s eye height into account. The first to document angular direction as a source of ground distance information were Wallach and O’Leary (1982; henceforth *WOL*). WOL developed a novel optical instrument to distort the apparent angular direction to an object on the ground (i.e., the “slope of regard”) without altering the perceived direction of the horizon, and showed that the apparent size of the object was appropriately altered by this manipulation. That is, compressing the apparent vertical angular deviation from straight ahead increased the perceived width of the object, consistent with the notion that the object was perceived as being farther away.

As we will explain below, WOL’s decision to measure perceived size led to an ambiguity as to whether perceived distance was actually affected by their optical manipulation. Here we report a replication in which we reconstructed their optical device and measured both perceived size (replicating the main conditions of their experiments) and perceived distance (extending their work). Although we now have independent reasons to believe that angular declination is an important distance cue that controls walking behavior and explicit distance perception (Li et al., 2013; Messing & Durgin, 2005; Ooi, Wu, & He, 2001), no one apart from WOL have previously directly manipulated perceived angular declination without altering the perceived horizon itself.

For example, Ooi, Wu, and He (2001) showed that adaptation to base-up prism goggles changed perceived distance (measured by walking behavior) in a manner consistent with a resetting of the perceived height of the (implicit) visual horizon. But using prisms to alter the apparent horizon also alters the apparent orientation of the ground plane (see Harris, 1974). Messing and Durgin (2005) showed that a subtle direct manipulation of the explicit visual horizon in a virtual environment (i.e., lowering it by 1.5°) also shifted perceived distance as predicted—both for explicit estimation and for a blindfolded walking task. But these manipulations both involve shifting the perceived horizon rather than rescaling perceived angles relative to the true horizon as WOL did. WOL’s rescaling of perceived angular declination is of particular current interest because of recent evidence that people may generally underestimate egocentric ground distance because their perceptual scaling of angular declination is already distorted (Durgin & Li, 2011; Li & Durgin, 2012; Li, Phillips, & Durgin, 2011). The device employed by WOL is a sort of embodiment of angular expansion/compression.

WOL described their device as “an analogue of a Galilean telescope of .7 power, composed of cylindrical, rather than spherical, lenses” (Wallach & O’Leary, 1982, p. 146). Our reproduction of their device used a custom, 3-D-printed plastic housing in conjunction with stock cylindrical lenses that are commercially available. A schematic diagram of the optical device is shown in Fig. 1. This device must be mounted horizontally to keep the perceived horizon correct.

**Fig. 1:**
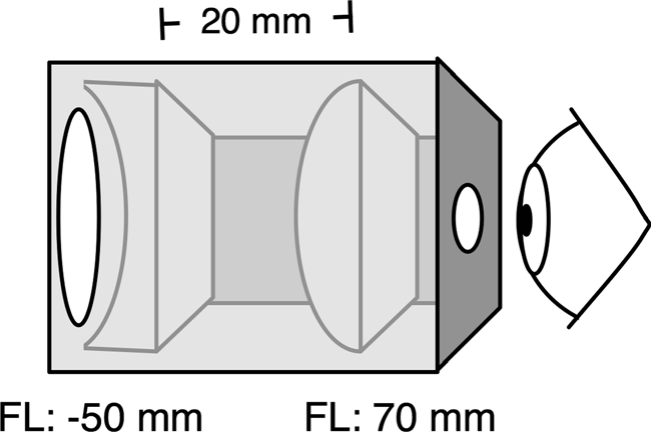
The lenses used for the reconstruction of Wallach and O’Leary’s (1982) cylindrical Galilean telescope were a concave cylindrical lens (used as the objective lens) with a focal distance of –50 mm and a convex cylindrical (eyepiece) lens with a focal length of 70 mm. Mounted 20 mm apart, they produced a collimated lens system with a vertical minification by a factor of 50/70 = .71

Two studies by Sedgwick and colleagues (Shah & Sedgwick, 2004; Tran & Sedgwick, 2008) on the effects of optical magnification (through a low-vision telescope) on perceived distance did not maintain a horizontal optical axis. Indeed, no effect on perceived distance was observed in their first study, in which the optical axis moved with the head (thus eliminating any optical directional bias during direct viewing of the target). In the second study, the optical axis was fixed, but inclined, and the effects found could be attributed to an optical offset of the apparent horizon, specified by texture cues alone (linear perspective). Neither study accomplished the goal of the present study—to offset perceived visual direction without offsetting the optical horizon.

Figure 2 shows how the geometry of vertical angular minification may lead to perceiving an object to be at a farther location along the ground. Changes in apparent distance will occur if angular declination (*γ*) is used to estimate ground distance. The vertical angular extent of the object (*ρ*) is also reduced, but the horizontal angular extent is unaffected by the cylindrical lenses, as is shown in Fig. 3.

**Fig. 2:**
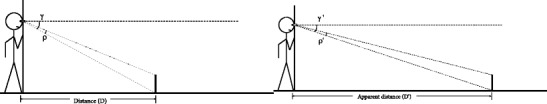
The right panel shows the optical effect of the lens system on the geometry depicted in the left panel. The angles *γ* and *ρ* are decreased, and the apparent distance based on *γ* should be increased

**Fig. 3:**
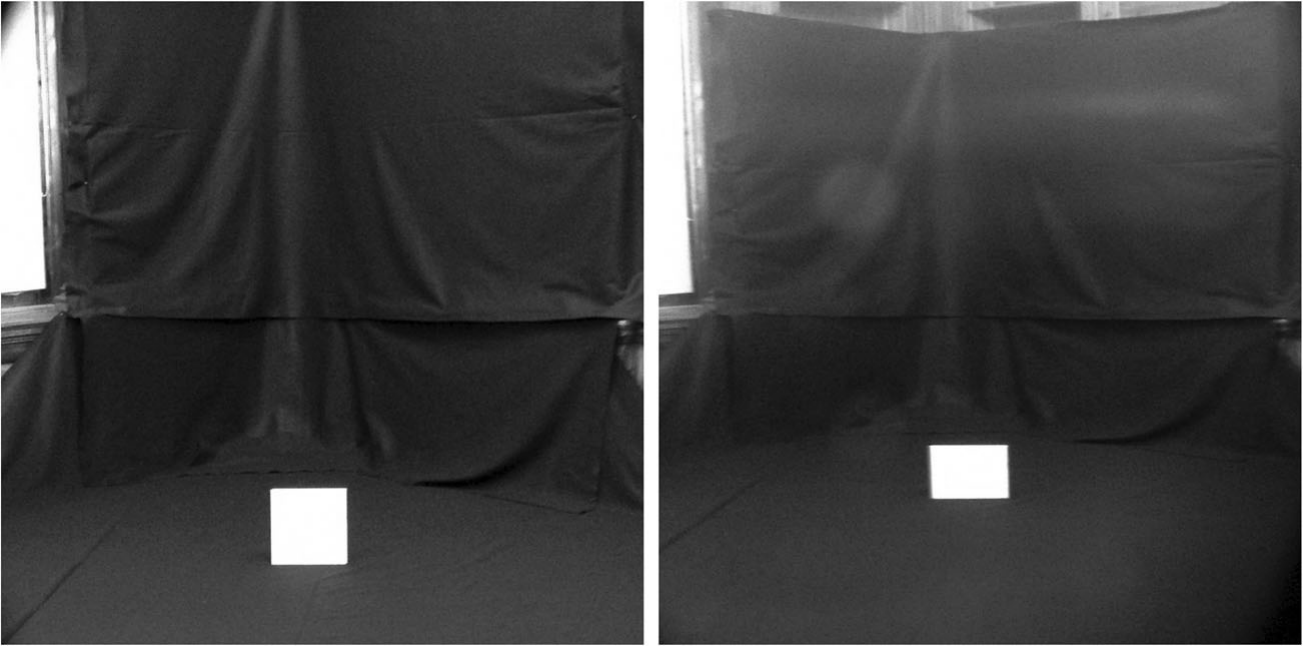
Photographs (cropped to about 24° × 24°) of the size stimulus viewed through the optical housing without lenses (left) and with the lenses (right). This pictorial representation suggests that the rectangle on the right appears to be farther away. For a situated observer (one viewing the scenes directly), the two rectangles seem equal in height but different in width

WOL found that the perceived width of an (actually square) object standing upright on the floor was increased when viewed through the lenses. They interpreted this as an implicit measure of an increase in the perceived distance for a situated observer: The angular horizontal extent of the object was unaffected optically; thus, the greater perceived width seemed to be due to an increase in the apparent viewing distance (see Fig. 3).

However, an alternative account of the change in perceived width could be based on the use of the *horizon ratio* (Rogers, 1996; Sedgwick, 1973, 1980): Upright objects at any distance on level ground cross the (implicit or explicit) horizon at eye height (Mark, 1987; Warren & Whang, 1987; Wraga, 1999). Thus, the height of an object standing on the ground could be expressed in eye-height units approximately as the ratio of its vertical angular extent relative to the angular extent between its base and the horizon (i.e., straight-ahead). Because the lens system developed by WOL did not change the horizon ratio, but did increase the apparent horizontal : vertical aspect ratio of the object, a change in the perceived width of the object might be expected on the basis of the unaltered horizon ratio in conjunction with the altered aspect ratio of the object. That is, if the object correctly appeared 28 cm high with or without the lens system, then it must have appeared much wider than 28 cm when the lenses altered its aspect ratio.

In the photographs of our own setup (with and without the lens system), shown in Fig. 3, there is no visual representation of the true horizon (which was higher than the horizontal seam shown in the hanging black felt). When looking at these images, the pictorial cue “height in the field” primarily provides an impression that the apparently rectangular surface is farther away than the square surface. However, when an observer is situated within the scene by looking through the lenses of the cylindrical Galilean telescope (which was adjusted to eye level for each observer) into the room, the implicit horizon is defined by the visually perceived eye level (VPEL), and the impressions of both size and distance appeared to us quite in accord with the claims of WOL. We therefore sought to replicate their original measure of perceived size and also to extend their work by adding direct measures of perceived distance in a second experiment.

## Experiment 1: Replication of the increase in perceived width with the compression of angular declination or “slope of regard”

WOL used a within-subjects design to measure the perceived height and width of an upright square object either with the lens system upright (compressing the vertical dimension) or turned sideways (so that the horizontal axis was minified), or with it absent. Their observations, though largely consistent with their hypothesis, showed large sequential dependency effects between the viewing conditions (as might be expected, given that the observers knew they were looking into the same room each time), so we adopted a between-subjects design and tested only the two main conditions, involving the presence or absence of vertical minification. The room that we used was a seldom-used classroom, with which very few of our participants were familiar, draped with black felt to obscure familiar size and texture information. During debriefing, all participants indicated they had not been aware of the optical distortion when viewing the room.

### Method

#### Participants

A total of 39 students (20 tested with lenses, 19 without) participated in exchange for candy. All were naïve to the manipulation and the hypothesis, and merely consented to make spatial judgments. The data of one participant run in the no-lens condition were excluded because of size estimates that were more than four standard deviations larger than those of the other participants.

#### Stopping rule

WOL had tested 36 participants across three counterbalanced conditions. On the basis of an estimated between-subjects effect size of nearly 1.0, with a desired power of .80, our goal was to run at least 36 participants across two between-subjects conditions over the limited period when we had access to the testing space (which was normally a classroom). Because signups for the experiment (and no-shows) were stochastic, we collected the data from slightly more than the desired 36 participants.

#### Materials and procedure

In addition to the lens system shown in Fig. 1, we used an identical housing without lenses for the control condition. One or the other housing was mounted in advance with its axis horizontal, in a mounting that could be quickly adjusted to the eye height of each observer. A 7 × 8 m room, depicted in Fig. 3, was draped in black felt in order to minimize ground texture and familiar size cues (WOL had used a large carpeted space with minimal ground texture) and was not visible to the participants except through our apparatus, which was designed to fill the entranceway. The visual target, a piece of white foam core (28 × 28 cm) stood vertically on the floor at a distance of 6 m along the ground from the observer’s eye position. Participants adjusted a retractable tape measure, viewed from the back, to match the apparent dimensions of the target, with the order of the width and height estimations varied between subjects within each condition. Note that WOL had used a visual target with similar dimensions and had used a telescoping rod as their matching stimulus. Participants were allowed to look freely back and forth between the (monocular) view of the room and the (binocularly viewed) tape measure. An experimenter recorded the participants’ estimates of both dimensions to the nearest ¼ in., which were later converted to centimeters. The entire procedure took only a few minutes from the time the participant gave consent.

The field of view (FOV) immediately available to the observer depended on the 3-D position of the pupil relative to the eyepiece aperture. From a fixed position near the aperture, the maximum FOV was an oval about 45° tall and 30° wide, as determined by photographs through the apparatus, but an additional 3° in each direction could be obtained by moving one’s vantage point relative to the eyepiece aperture. For someone wearing spectacles (which might enforce a greater distance between the pupil and the viewing aperture), the vertical FOV could have been somewhat reduced, but the overall position (up/down and left/right) of this apparent window could also be shifted by very slight adjustments of posture, still allowing a good view of the target in the scene. Thus, with the telescope fixed at horizontal, an observer could either see or scan over an effective vertical FOV of at least 50°. This effective FOV was essentially the same even when the lenses were removed.

The compression of the angular declination to the base of the size stimulus necessarily meant that more of the floor was visible in front of the object when the lenses were in. But past studies have suggested that the amount of visible floor is not a strong cue to egocentric distance (e.g., Creem-Regehr, Willemsen, Gooch, & Thompson, 2005). Moreover, the amount of visible floor depended somewhat on where the observer held his or her eye relative to the eyepiece. Note that, like WOL, we sought to minimize texture information on the floor by using a fairly uniform floor covering.

#### Manipulation check

The horizontally aligned optics of the instrument were intended to maintain the same visually perceived eye level (VPEL) optically. Moreover, even the minimal ground texture that was available (seams and folds in the fabric on the floor) would be consistent with the correct vanishing-point horizon through the optics. To confirm that VPEL was not altered by looking through the optics, ten additional participants were tested later using a reconstruction of the same experimental environment. Forty trials of a forced choice staircase procedure were conducted both with and without the lens, in counterbalanced order, using an oval of light (2 cm high and 4 cm wide) projected onto the black felt at the far corner of the (fully illuminated) room as the test stimulus. Perceived VPEL did not differ as a function of the lenses being present (*M* ± *SD*: 0.2° ± 0.7°), *t*(9) = 0.86, *p* = .41, though the perceived VPEL was slightly lower than the true horizontal across both conditions (*M* ± *SD*: –1.0° ± 0.9°), *t*(9) = 3.24, *p* = .0102. This shows that the present optical manipulation (unlike prism adaptation and virtual horizon manipulations) did not substantially influence the apparent location of the implicit horizon in the experimental environment.

### Results and discussion

**Fig. 4:**
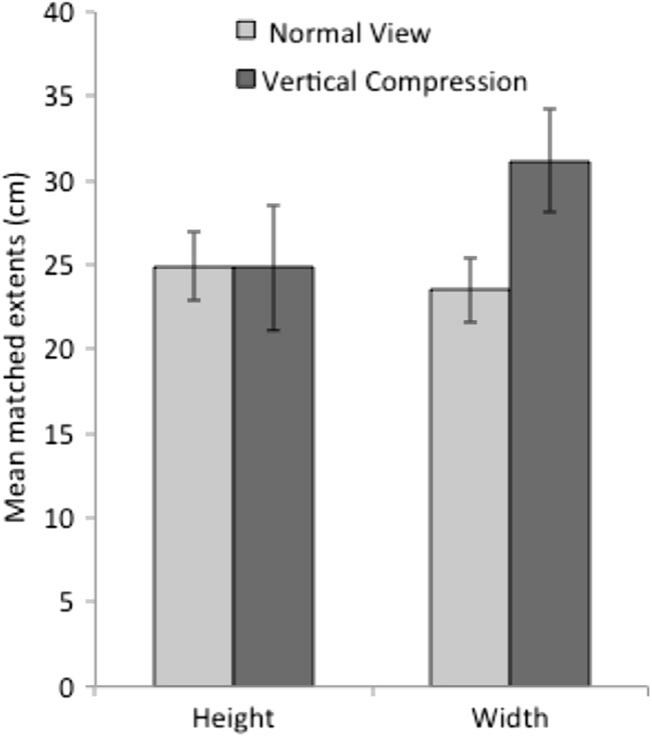
Results of the two viewing conditions of Experiment 1. Standard errors of the means are shown

The mean estimates of the height and of width of the target are shown in Fig. 4 for each viewing condition. As expected, the estimates of vertical extent (*M* = 25 cm) were identical across viewing conditions, but estimates of the width in the lens condition (31 cm) were reliably higher than those without lenses (24 cm), *t*(36) = 2.05, *p* = .0474, Cohen’s *d* = 0.68. For those without lenses, matches to the width of the shape were smaller by 6 % than those to the height, *t*(17) = 2.45, *p* = .0252, *d* = 0.56, consistent with the presence of a 6 % horizontal–vertical illusion (HVI: Finger & Spelt, 1947). Among those viewing the scene through the vertical compression lenses, the matched widths were reliably larger (by 26 %) than the heights, *t*(19) = 4.36, *p* = .0003, *d* = 0.98. If compensation for a 6 % HVI is included, the ratio of width to height is 33 % (which also corresponds to the ratio of the judged widths between the two groups).

On the basis of a ground distance of 6 m, the expected increase in perceived viewing distance for typical eye heights would be 41 %. The angular change in projected aspect ratio would be by 40 %. Thus, the present results, which replicate the findings of WOL for these conditions, are quantitatively consistent with either a partial increase in perceived distance (e.g., by about 33 %), based on the compressed angular declination to the base of the target, or with a partial response to the change in perceived aspect ratio in conjunction with a stable estimate of the height of the target based on a constant horizon ratio. Experiment 2 was designed to further test whether perceived distance was altered by the optical manipulation of angular declination.

## Experiment 2: Extension to the direct measurement of perceived distance

Our own perceptual experience with vertical compression through the lenses suggested the presence of a change in perceived distance even for a situated observer (i.e., not simply as a pictorial cue). But WOL did not measure perceived distance directly, and their size results are now known to be ambiguous with respect to whether perceived size alone, rather than perceived distance, was altered. To try to disambiguate the situation and test whether we could document the change in perceived distance that we ourselves observed through this unusual viewing apparatus, we conducted a second experiment in which we measured perceived distance both explicitly (by verbal estimation) and implicitly (by a beanbag toss). In addition, we laid the target flat on the ground in order to minimize the relevance of the horizon ratio, while retaining angular declination as a source of distance information.

### Method

#### Participants

A total of 44 new students participated. They were divided among four between-subjects conditions crossing our optical manipulation (vertical compression lenses present or not) and the type of measure taken (verbal report or thrown distance). Because of a randomization fluke, 14 of the 22 people assigned to the no-lens condition were assigned to the throwing task. Eleven people were in each of the lens-present measurement conditions.

#### Stopping rule

Faced, again, with limited-duration access to the testing space (the same classroom), our goal was to obtain at least 40 participants within the time window available. Because signups (and no-shows) for the experiment were stochastic, we collected data from slightly more than the original goal of 40 participants.

#### Procedure

**Fig. 5:**
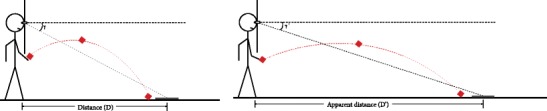
Setup for the throwing task of Experiment 2, illustrating the expected effects of the lenses on throwing both without (left) and with (right) the lenses in place.

The setup was similar to that used in Experiment 1, except that the target was flat on the floor and a low opening was established for underhand throwing beneath the occluding screen surrounding the lenses, as is depicted in Fig. 5. Participants either made a single beanbag toss, trying to “hit” the target (see Durgin, DeWald, Lechich, Li, & Ontiveros, 2011), or gave a single verbal estimate in metric or English units. Because participants made the toss while looking through the viewing aperture, their physical throwing motion was constricted by both the near immobilization of their head and the physical limits on their ability to swing their arm forward, as a result of the apparatus. The distance to the initial landing location of the beanbag was measured to the nearest centimeter by an experimenter using a laser measuring device.

### Results and discussion

**Fig. 6:**
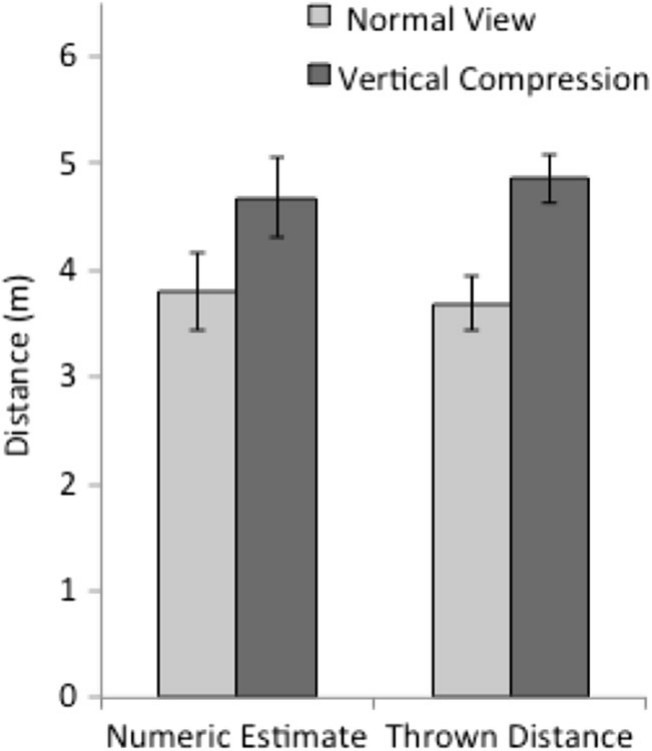
Results of all four conditions of Experiment 2. Standard errors of the means are shown

The mean estimates of distance and the mean thrown distances are shown in Fig. 6 for each viewing condition. For the throwing task, the distances thrown were reliably farther for those viewing the scene through the vertical compression lenses (4.9 m) than for those without lenses (3.7 m), *t*(23) = 3.30, *p* = .0031, Cohen’s *d* = 1.38, for a distance ratio of 1.32, which is essentially identical to that implied by the 33 % increase in width estimation observed in Experiment 1. Relatively short throws in both conditions may have resulted from the physical constraints on the throwing action.

The effect of the lenses on verbal distance estimation was in the same direction (ratio of 1.23), but it was not statistically reliable, *t*(17) = 1.62, *p* = .12, Cohen’s *d* = 0.78. Explicit distance underestimation in the no-lens condition was by a factor of .63, which is about .9 lower than is typically found (e.g., .7–.9; Loomis & Philbeck, 2008). This may have been induced by the reduced FOV provided by the viewing apparatus (see Wu, Ooi, & He, 2004).

In summary, the results of the throwing task seem to unambiguously indicate a change in perceived distance that is quantitatively consistent with the change in perceived width measured in Experiment 1. This provides supporting evidence that the perceptual experience of distance may indeed have been the source of the bias in perceived width in Experiment 1.

## General discussion

Whereas it is becoming fairly well established that angular declination (or gaze declination or “slope of regard”) might strongly contribute to the perception of egocentric ground distance for situated observers (Li et al., 2011; Messing & Durgin, 2005; Ooi et al., 2001), direct manipulations of angular declination have generally involved also altering the location of the (implicitly or explicitly) perceived horizon by means of either prism adaptation (Ooi et al.) or virtual environments (Messing & Durgin). Using the direct optical manipulation of perceived angular declination introduced by WOL for a situated observer, we have replicated their observation that perceived size is affected in a manner consistent with a distortion of perceived distance, and we have extended their observation by showing that even direct measures of perceived distance show evidence of distortion that is quantitatively consistent with that observed for size.

Investigations of size perception (using much larger objects than the one used here) have been conducted using false (raised) floors that were intended to affect horizon-ratio scaling (e.g., Warren & Whang, 1987; Wraga, 1999). Such a manipulation would also have affected the effective angular declination to the base of the object, though typically the magnitude of the angular change would have been fairly small in these studies. Among these studies, only Warren and Whang reported directly measuring perceived distance. They reported a null effect on verbal estimates of distance, whereas they found that passability (implicit width) judgments were affected by their false-floor manipulation. Wraga, in contrast (see also Bingham, 1993), concluded that explicit estimates of horizontal extents were not affected in the same way by the horizon ratio as were estimates of vertical extents. She noted that, for a mobile observer, retinal aspect ratio was more weakly related to the true aspect ratio than was horizon-relative height to the true height. Wraga speculated that the difference between her data and those of Warren and Whang regarding perceived width may have been due to the type of measure (i.e., explicit rather than affordance-based). Our data support a refinement of that view.

In directly manipulating angular declination, we replicated WOL, in that we found that explicit estimates of width were affected by a change in angular declination, but we also found that implicit estimates of perceived distance (thrown distance) showed reliable effects, whereas verbal estimates showed only a trend. The implicit width judgments used by Warren and Whang (1987) may have been more sensitive than were the verbal estimates of distance they had collected in their between-subjects design, and more sensitive than the width estimates collected by Wraga (1999). Perceptual judgments at an affordance boundary might be more sensitive because they are made relative to a categorical boundary (e.g., Bertamini, Yang, & Proffitt, 1998). In contrast, verbal reports of distance measured between subjects suffer problems of between-subjects scaling variance. Messing and Durgin (2005), using a within-subjects design, found reliable effects of a subtle horizon manipulation on verbal estimates of distance when they considered second-order representations of verbal distance estimates: They computed the exponents of the power function for each observer under each viewing condition (see Durgin & Li, 2010, for discussion). Using such an analysis, Messing and Durgin found similar effects of a subtle horizon shift on explicit verbal distance estimation and on a distance-walking task conducted with a different set of participants. In other words, even for a subtle shift in angular declination, both explicit (verbal estimation) and implicit (walked distance) measures of perceived distance can detect the effect of angular declination on perceived distance when scaling variance is made irrelevant. The effects measured in false-floor manipulations may depend on the details of other information available in the scene, as well as on the specific measures used. Our present data suggest that subtle perceived distance effects might well be present in false-floor manipulations, even though they have not been previously detected.

Wallach and O’Leary (1982) proposed that the slope of regard (i.e., angular declination) was a learned distance cue based on correlational experience with other cues (see Haijiang, Saunders, Stone, & Backus, 2006), and that it might be promoted as a cue when in conjunction with other cues including a standing posture and possibly walking. A clear advantage of this simple directional cue is that it can be based on monocular information and on otherwise reduced visual information (see, e.g., Gajewski, Philbeck, Pothier, & Chichka, 2010; Philbeck & Loomis, 1997), and it has consistently been shown to affect walked distance (e.g., Ooi et al., 2001). WOL emphasized that the use of this cue, even when the observer is situated in an environment, must be probabilistic, because it requires some estimate of eye height relative to the viewing surface (thus the importance of a standing posture). We note that it should also require an assessment of the ground surface orientation. Perceived angle of elevation has recently been implicated in the control of shooting action (i.e., distance perception) in basketball (de Oliveira, Oudejans, & Beek, 2009). In support of WOL’s learning perspective, this represents another context in which a fixed eye-to-hoop distance relationship can be exploited by processes of angular cue recruitment for action control.

It seems significant from a learning perspective that explicit reports of perceived visual direction (angular elevation and declination) show large systematic biases (Durgin & Li, 2011; Li & Durgin, 2009). These angular biases seem to correspond to observed linear-extent biases in the perceived layout of space, as measured by perceptual matching. For example, when asked to position themselves the same distance from a pole as the pole is high, observers set themselves much too far away (Durgin, Leonard-Solis, Masters, Schmelz, & Li, 2012; Higashiyama & Ueyama, 1988; Li et al., 2011). The geometry of their behavior can be explained quite well by assuming that perceived angular direction in pitch is misscaled (i.e., exaggerated) relative to actual deviations from the horizontal (Li et al., 2011). The implied angular misscaling is by a factor of 1.5, which is precisely consistent with explicit biases in judged pitch direction measured independently using a variety of verbal and nonverbal methods (Durgin & Li, 2011). Because well-practiced actions, such as walking, are evidently calibrated to this misscaling (Li et al., 2013; Loomis, da Silva, Fujita, & Fukusima, 1992), there appears to be no contradiction between the evidence that perceived angular declination is a powerful source of information about distance, as supported by the present study, and the evidence that angular elevation and declination may also be systematically misperceived. Durgin and Li (2011) proposed that the misscaling of angular declination is an informationally efficient coding strategy employed to maintain greater coding precision in the representation of angular direction. From this perspective, one driving force behind angular cue recruitment might be said to be gains in perceptual (i.e., coding) precision for the control of action. That is, an exaggerated coding of angular deviations from straight-ahead might tend to increase the advantages to be gained by learning to use angular declination as a source of information about ground distance. In any case, the possible status of angular declination as a learned distance cue remains an important impetus for future research.

The use of Galilean telescopes with cylindrical lenses to alter the perceived angular direction to an object on the ground provides opportunities and challenges for researchers. An advantage of the telescopes is that they do not alter the perceived orientation of the ground plane, as do base-up and base-down prisms (see Harris, 1974). However, the telescopes would not work as intended here unless their optical axes remained horizontal (see Shah & Sedgwick, 2004): If worn as a goggle that moved with the head, the apparent angular elevation/declination would be compressed relative to the orientation of the goggle, with the result that head movements (in the pitch axis) and eye rotations in pitch within the head would produce different perceptual experiences of visual direction. In the short term, this produces an obvious deformation of the scene with head movements. Moreover, when two of these telescopes are mounted side by side to produce a stereoscopic goggle, a clear conflict can be observed between the distance information provided by (distorted) angular declination and that provided by binocular information, including vergence (which is unaffected by the goggles). In our informal observations, vergence, which is a more certain cue, appears to “win” during short-term exposures.

We have not yet measured the effect of adapting observers to the continuous use of such a stereoscopic vertical-compression goggle, but one would expect that it might be possible to adapt both to the discrepancy between the apparent visual directions based on angular declination and on changes in head pitch, and to the discrepancy between the egocentric distances signaled by vergence and by apparent angular declination. Such experiments would be very much in the spirit of Wallach’s earlier work on adaptation to visual cue conflict (e.g., Wallach & Frey, 1972; Wallach & Karsh, 1963; Wallach, Moore, & Davidson, 1963), as well as of the subsequent study of perceptual adaptation and learning based on such conflict (e.g., Fisher & Ebenholtz, 1986; Priot, Laboissière, Sillan, Roumes, & Prablanc, 2010).
